# Exploration of heterogeneous catalyst for molecular hydrogen ortho‐para conversion

**DOI:** 10.1002/EXP.20230040

**Published:** 2023-12-14

**Authors:** Hideki Abe, Hiroshi Mizoguchi, Ryuto Eguchi, Hideo Hosono

**Affiliations:** ^1^ Center for Green Research on Energy and Environmental Materials National Institute for Materials Science (NIMS) Tsukuba Ibaraki Japan; ^2^ Graduate School of Science and Technology Saitama University Saitama Japan; ^3^ Research Center for Materials Nanoarchitectonics (MANA) National Institute for Materials Science (NIMS) Tsukuba Ibaraki Japan; ^4^ Faculty of Pure and Applied Sciences University of Tsukuba Tsukuba Ibaraki Japan; ^5^ MDX Research Center for Element Strategy International Research Frontiers Initiative Tokyo Institute of Technology Yokohama Japan

**Keywords:** catalyst, hydrogen, hydrogen economy, hydrogen liquefaction, ortho‐para conversion

## Abstract

Molecular hydrogen (H_2_) ortho‐para conversion (O/P conversion) proceeds slowly at low temperatures accompanying a heat release. Thus, catalysts for accelerating this conversion rate are highly demanded in terms of the storage and utilization of liquid H_2_. The catalysts for this purpose are experimentally screened by examining a broad range of materials covering magnetic, non‐magnetic, metallic, and nonmetallic oxides. The primary conclusions obtained are summarized below. (1) active materials are required to be non‐metallic and to bear the cations with ionic radii smaller than the bond length of H_2_. (2) Metallic materials have almost no activity irrespective of with or without magnetism (3) The activity of materials belonging to (1) is largely enhanced when the constituting cation has a magnetic moment. In addition, there is a class of materials for which the activity is distinctly enhanced just upon substitution by the foreign ions.

## Graphical abstract

The catalysts for nuclear spin conversion of molecular hydrogen (ortho‐para H_2_ conversion), are categorized into four groups in the ascending order of activity at 77 K; Group 1: metallic materials and most of the oxides containing low‐valence metal cations; Groups 2 and 3: metal oxides comprising high‐valence cations; Group 4: metal oxides and hydroxides that consist of magnetic metal cations.

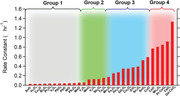

## 1 INTRODUCTION

**FIGURE 1:**
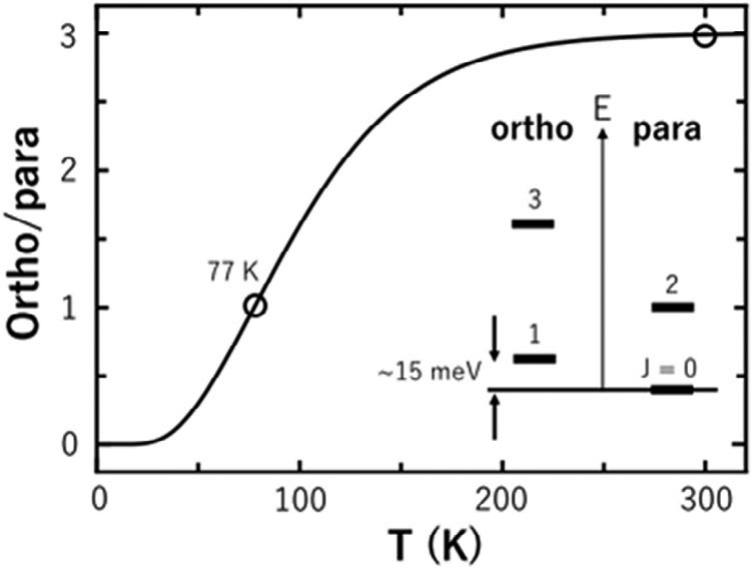
Equilibrium ratio of ortho/para H_2_ concentration. Inset shows the energy diagram for molecular hydrogen.

The dominant role of hydrogen in sustainable future energy or hydrogen economy, is widely accepted based on its excellent nature as an energy source for alternative fuel.^[^ ^1^, ^2^ ^]^ It is a scientific consensus that four key technologies are required for the promotion of the hydrogen economy: hydrogen production, transportation, storage, and utilization.^[^ ^3^ ^]^ The cooling and liquefaction of molecular hydrogen (H_2_) enable the storage of large quantities of hydrogen fuel.^[^ ^4^, ^5^ ^]^ However, there is a technical obstacle to overcome for the industrial use of liquefied H_2_: There are two isomers of molecular H_2_, ortho H_2_ with nuclear spin (*J*) of 1 and para H_2_ with *J* = 0 ‐forms. In the thermal equilibrium state, ortho H_2_ and para H_2_ occupy the rotational ground states of *J* = 1 and *J* = 0, respectively, and the O/P ratio is 3 near the ambient temperature, but this ratio becomes 0 (only para H_2_ exists) at the liquefied temperature of H_2_, 20 K (Figure 1). Since ortho H_2_ has a non‐zero moment of *J* = 1, it relaxes to the para‐state in the condensed state where the molecular separation is 0.37 nm at low temperatures at a time constant of ∼100 h at 20 K (O/P conversion). The rotational energy released from the O/P conversion corresponding to *J* = 1→0 is 15 meV, which is larger than the evaporation energy (∼12 meV) of liquefied H_2_ (Figure 1, inset). As a result, ∼55% of liquefied H_2_ is lost through evaporation by the O/P conversion heat when conventional H_2_ gas with an O/P ratio of 3 is equilibrated at 20 K. Such an H_2_ evaporation loss is called “boil‐off.” To avoid the boil‐off, catalysis to promote the O/P conversion is needed to store liquid para H_2_ with *J* = 0.

In 1933, E. Wigner reported a theoretical consideration on the O/P conversion and proposed that this conversion is induced on the adsorbed H_2_ on the magnetic materials by an inhomogeneous magnetic field arising from the electron spin magnetic dipole moment.^[^ ^6^, ^7^, ^8^ ^]^ The interaction is proportional to *μ* ^2^/*r* ^6^, where *μ* is the magnetic moment of the magnetic ion and *r* is the collision distance. Considering Wigner's theory, various O/P catalysts have been developed so far. Much effort has been devoted to the development of new catalysts since the 1960s.^[^ ^9^, ^10^, ^11^, ^12^, ^13^ ^]^ Currently, 3*d*‐transition metal oxides such as Cr_2_O_3_ and hydrated iron(III) oxide (Fe_2_O_3_•*n*H_2_O) are used as catalysts for this purpose.^[^ ^14^, ^15^, ^16^, ^17^, ^18^ ^]^ However, it has continued to seek more efficient catalysts as well as understanding the effective conversion mechanism until recently.^[^ ^19^, ^20^, ^21^, ^22^ ^]^

Since then, it has been believed that this conversion is not induced on the surface of diamagnetic materials. However, in the 1980s, it was found that this conversion occurs even on various nonmagnetic materials, such as amorphous ice.^[^ ^23^, ^24^ ^]^ Recently, the conversion of H_2_ confined into the nano space of metal‐organic framework (MOF) has gained attention.^[^ ^25^, ^26^ ^]^ So far, fundamental research on the O/P conversion on solid surfaces has been performed employing clean materials, surfaces and physical techniques.^[^ ^24^ ^]^ According to these researches, the O/P conversion is induced by three origins: magnetic fields,^[^ ^6^, ^7^, ^8^ ^]^ charge transfer,^[^ ^27^, ^28^, ^29^ ^]^ and electric fields.^[^ ^24^ ^]^ Although each mechanism appears to be valid for the materials system examined, we think it is pivotal at the present stage for rational catalyst design to get a rough but comprehensive image of the concrete catalytic materials.

In this work, we explore the O/P conversion at 77 K for a wide range of solid materials covering magnetic/non‐magnetic and metallic (no opened band gap)/semiconducting materials and classify these materials into different types depending on the conversion activity. As a consequence, the experimental results may be classified into four types, and each type is featured by the mechanism except for several exceptions. The essential factor for the high catalytic activity is not magnetism but bearing the cation with small ionic radii in the non‐metallic materials, to our surprise.

## 2 EXPERIMENTAL

### 2.1 Catalyst materials

We investigated a wide range of solid materials covering magnetic/non‐magnetic, metallic (no opened band gap) and semiconducting materials. These powder samples were purchased from the manufacturers (Sigma‐Aldrich, Alfa Aesar, Kishida Chemical, Kojundo Chemical Laboratory, Fruuchi Chemical, or Furuya Metal) or made by ourselves by gas atomizing or gas reduction of precursors. The particle size of most of the catalysts was in the range of 1 to 10 μm, except for some nanoparticulate catalysts, including SnO, Sn_3_O_4_, NiO, CuO, and Pr‐doped CeO_2_, whose particle size was smaller than 100 nm. See Figures S1 and S2, Supporting Information, for powder X‐ray diffraction (*p*XRD)  data for the catalysts.

**FIGURE 2:**
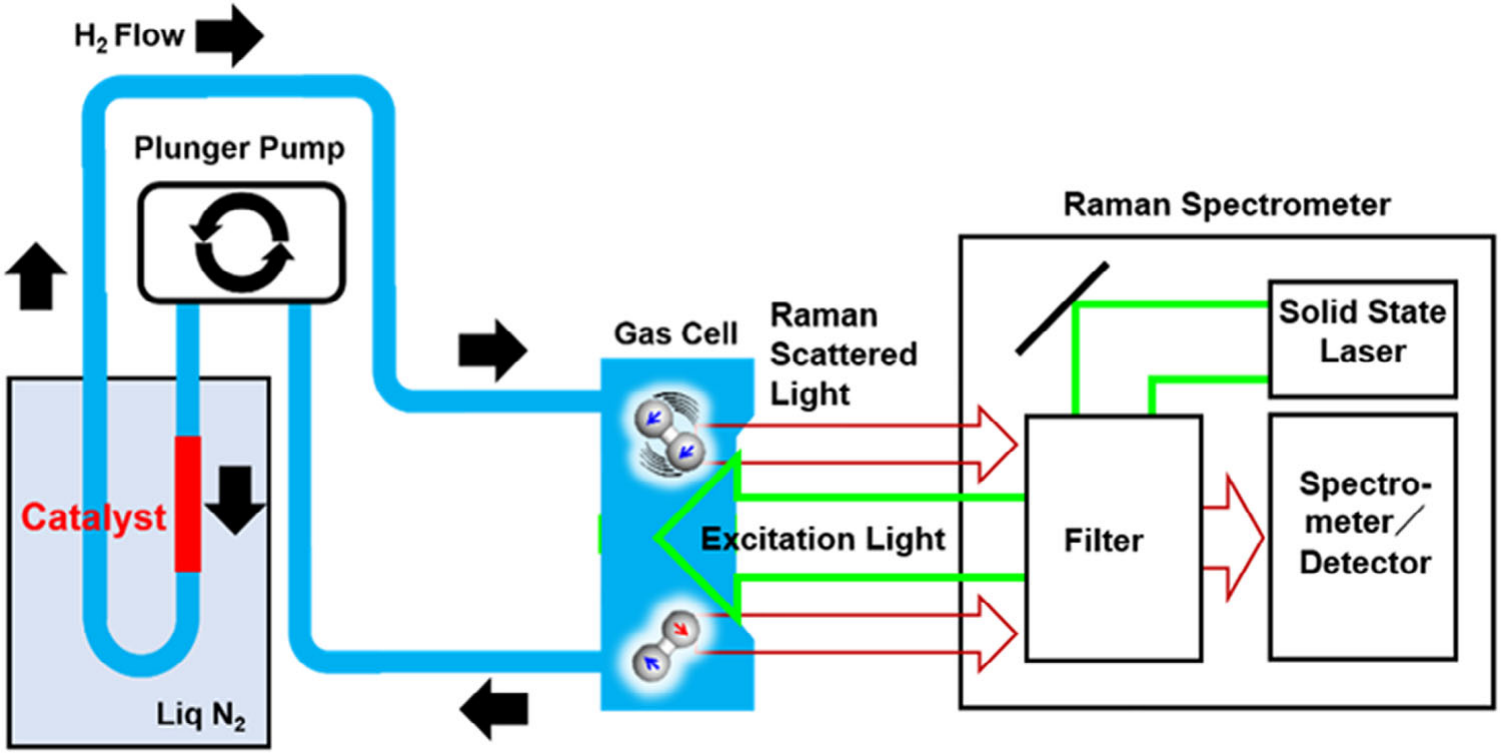
Experimental setup for the catalytic O/P conversion measurement.

The O/P conversion activity of various catalyst materials was evaluated at 77 K using a batch reactor equipped with a plunger pump and a gas‐tight cell, respectively for gas circulation and Raman spectroscopy (Figure 2). H_2_ gas (99.9%) as purchased was used without further purification. An aliquot of 100 mg of catalyst particles (particle size < 10 μm) was loaded in a glass‐made sample tube with an inner diameter of 6 mm. The sample tube was attached to the batch reactor, evacuated down to 10 Pa, and backfilled with H_2_ gas up to 80 kPa. The H_2_ gas was repeatedly passed through the catalyst layer and the gas cell in sequence, being monitored with a Raman spectrometer (JASCO RMP‐510) of the population of ortho‐ and para H_2_.

**FIGURE 3:**
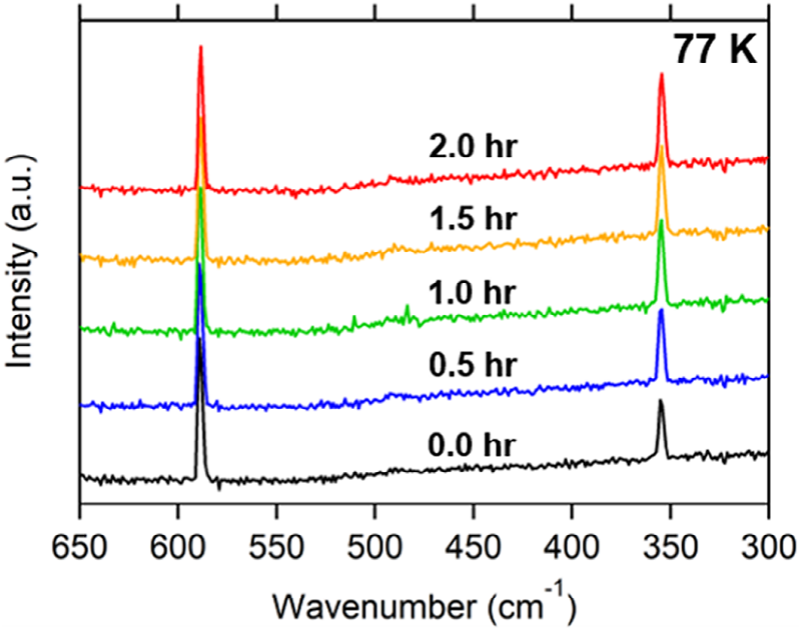
Time evolution of Raman spectra for the O/P conversion over Fe_2_O_3_ catalysts.

Figure 3 shows a series of Raman spectra acquired at 77 K at different duration times after the circulating H_2_ gas was subjected to an iron oxide (Fe_2_O_3_) catalyst. The volume fraction of para H_2_ was calculated as 29 ± 1.0% from the intensity ratio of the major Raman peaks at 354.4 and 588.4 cm^−1^ that correspond to the *J* = 0 to *J* = 2 and *J* = 1 to *J* = 3 transitions (see the inset of Figure 1), respectively.^[^ ^30^ ^]^ The evaluated fraction was close to 25%, which is theoretically expected at room temperature from the Boltzmann statistics. The para H_2_ fraction increased to reach 39 ± 1.0%, 4 h after the catalysis was started by immersing the sample tube into liquid nitrogen. By contrast, the O/P conversion was hardly promoted when aluminum oxide (Al_2_O_3_) was used as the catalyst. The para H_2_ fraction remained around 25% even 5 h after the catalysis was started, showing that the O/P conversion activity of Al_2_O_3_ is negligibly low compared to that of Fe_2_O_3_ in this catalysis condition.

## 3 RESULTS

**FIGURE 4:**
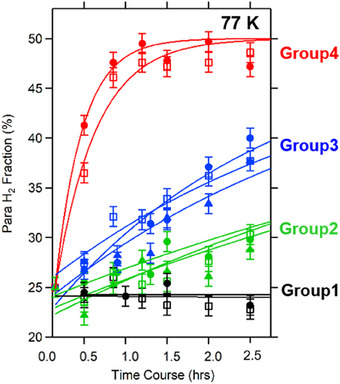
Trends of the O/P conversion at 77 K over different catalysts. The black, green, blue, and red curves are assigned to the materials of groups 1, 2, 3, and 4, respectively. Red‐filled circles and open squares correspond to Mn_3_O_4_ and CoO, respectively. Blue‐filled circles, open squares, and filled triangles correspond to SnO_2_, Ho_2_O_3_, and Sn_3_O_4_, respectively. Green‐filled circles, open squares, and filled triangles correspond to Sb_2_O_5_, V_2_O_5_, and Ta_2_O_5_, respectively. Black‐filled circles and open squares correspond to FeO and metallic Gd_5_Si_3_, respectively. The error bars were calculated as a standard deviation of the background spectrum acquired by filling the sample tube with no catalyst.

Figure 4 shows the trend of the O/P conversion catalysis at 77 K for different materials, including metal and single metal oxides (*A*O*_x_*), which are categorized into four groups. The first is a group of materials that exhibit no finite activity toward the O/P conversion (group 1, black curves in Figure 4). This group involves all of the metallic materials and most of the oxides containing low‐valence metal cations. The para H_2_ fraction stayed around the initial value of 25% over hours even though H_2_ gas repeatedly passed through the metal catalysts, including bismuth (Bi), gold (Au), platinum (Pt), or intermetallic compounds such as ErAl_2_, GdAl_2_, or HoB_2_. The metal oxides containing hollow or filled *d*‐orbitals such as Cu_2_O or ZnO were as inert as the metal catalysts. None of the low‐valence metal oxides, such as Mn^2+^O, Fe^2+^O, Ni^2+^O, Sn^2+^O, Pb^2+^O, V^3+^_2_O_3_, In^3+^_2_O_3_ or Bi^3+^_2_O_3_ efficiently promoted the O/P conversion. The second group consists of metal oxides that contain high‐valence cations such as V^5+^_2_O_5_, Mn^4+^O_2_, Ta^5+^_2_O_5_, and Sb^5+^_2_O_5_ (group 2, green curves in Figure 4).

The group 2 materials exhibited finite O/P conversion activity; the para H_2_ fraction monotonously increased from 25%, showing a tendency to saturate toward 50%. Most of the group 2 oxides comprise non‐magnetic metal ions without *d*‐electrons (V^5+^, Y^3+^, Ta^5+^, Sb^5+^). The third group involves metal oxides comprising high‐valence metal ions such as Mn^3+^_2_O_3_, Cu^2+^O, Zr^4+^O_2_, Sn^4+^Sn^2+^_2_O_4_, Sn^4+^O_2_, Ho^3+^_2_O_3_ and Gd^3+^_2_O_3_ (group 3, blue curves in Figure 4). The group 3 materials are similar in ionic and catalytic nature to the group 2 materials, yet showed superior catalytic activity. The last group involves some of the metal oxides and hydroxides that consist of magnetic metal cations (group 4, red curves in Figure 4). Mn^2+^Mn^3+^_2_O_4_^[^ ^31^ ^]^ and Co^2+^O showed much higher catalytic activity than any other materials belonging to groups 1, 2 or 3. The O/P conversion over Mn_3_O_4_ and/or CoO was so fast that the para H_2_ fraction reached the theoretical maximum of 50%, within a half hour. Cerium dioxide (CeO_2_) exhibited higher activity even than Mn_3_O_4_ or CoO. Mn_3_O_4_ and CoO have been known as semiconductors having a bandgap of 2.8 and 2.1 eV, respectively.^[^ ^32^, ^33^ ^]^ Moreover, the inherent activity of CeO_2_ was significantly improved when the tetravalent Ce^4+^ was partially substituted with trivalent Gd^3+^ as gadolinium‐doped ceria (Gd: CeO_2_).

## 4 DISCUSSION

**FIGURE 5:**
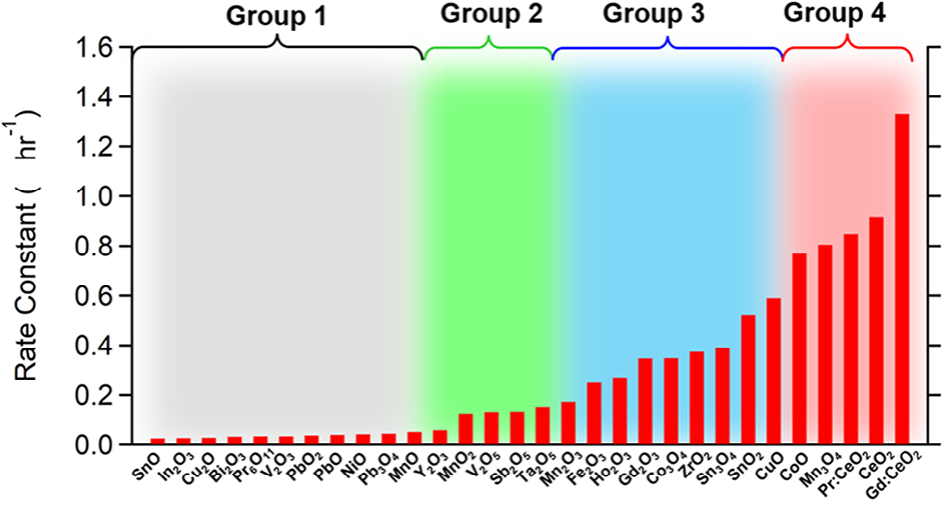
Rate constants of the O/P conversion over representative catalyst materials. The rate constants were obtained by numerical fitting to the experimental data using an exponential function (see Figure S3, Supporting Information, and the caption for details on the numerical fitting).

First, we discuss the effect of an electric field which works in nonmagnetic ionic compounds categorized into group 3. H_2_ molecules are modulated in nuclear spins through an interaction with the electrons of ions constituting the surface when weakly adsorbed on the catalyst surface retaining the H‐H bond (physisorption). It is acknowledged that quantum transition in the total spin momentum of H_2_ nuclei from *J* = 1 to *J* = 0, namely the O/P conversion, can be promoted especially when the physisorbed H_2_ molecules are subjected to localized electric fields with high intensity and spatial anisotropy.^[^ ^6^ ^]^ The effect has been considered to be key to realizing the conversion on MOF or amorphous ice.^[^ ^23^, ^25^ ^]^ Such anisotropic electric fields are scarcely formed over the metal surface, where the local charge is evenly screened and smoothed by itinerant electrons. The screened, isotropic electric fields can hardly promote the nuclear spin transition, resulting in the very low O/P conversion activity of metallic materials (Figure 5).

**FIGURE 6:**
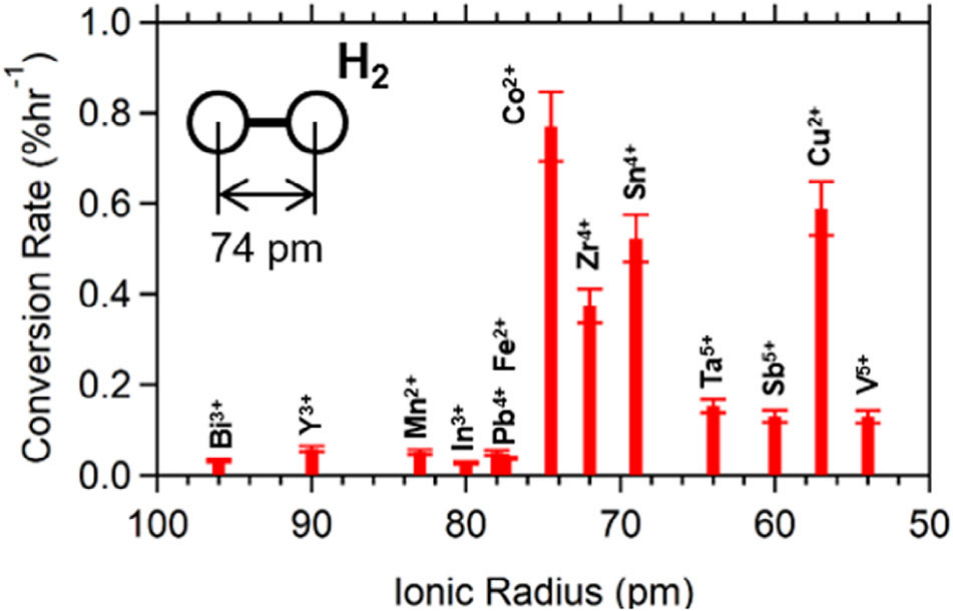
Relation between the O/P conversion activity of single metal oxides and the radius of the constituent metal ions.

Here, we attempt to compare the size‐matching between H_2_ and the catalyst surface. The ionic radius proposed by Shannon^[^ ^34^ ^]^ is utilized as a measure. The ionic radii for Bi^3+^ (96 pm), Y^3+^ (90 pm), and In^3+^ (80 pm) are larger than the bond length of H_2_: 74.1 pm (Figure 6). The ionic radius of Pb^4+^ in PbO_2_, 78 pm, is also larger than 74.1 pm. As already addressed in the experimental section, the low‐valence metal oxides such as Bi_2_O_3_, Y_2_O_3_, MnO, In_2_O_3_, and FeO show much low O/P conversion activity. The activity of PbO_2_ is as low as that of the other low‐valence metal oxides. The large‐sized metal ions are not favorable to offer sufficiently strong electric fields to the H_2_ molecules, which results in low O/P conversion activity. Unlike the metal oxides comprising large metal ions, the metal oxides composed of small metal ions (ionic radii < 74.1 pm) exhibit high O/P conversion activity (Figure 6).

**FIGURE 7:**
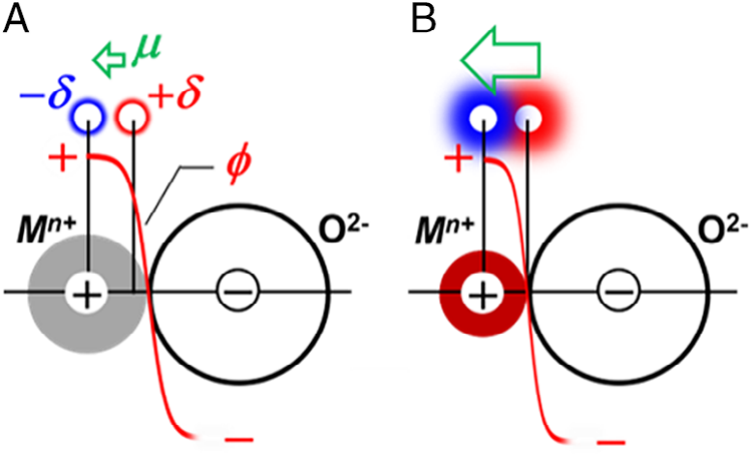
Polarization of H_2_ molecules by anisotropic electric fields between the oxygen‐ and metal ions. The steep gradient in the electric potential (*φ*) at the border of the metal‐ and oxygen ions polarizes the H_2_ molecule (± δ). The atomic environment around the small metal (*b*) ion is more favorable than the large metal ion (*a*) to develop electric dipole moments (*μ*).

The H_2_ molecules physisorbed on the small metal ions are strongly polarized to interact with the anisotropic electric field, resulting in promoted O/P conversions. Over the surface of the metal oxides belonging to group 1, such as Mn^2+^O^2−^, Fe^2+^ O^2−^, Sn^2+^ O^2−^, and Pb^2+^ O^2−^, there are distributed local extrema of electric potentials at each of the surface ions. The electric potential abruptly changes the polarity at the ion boundary to develop an electric field with high anisotropy (Figure 7). However, low‐valence metal ions are large: the ionic radii of Fe^2+^ (HS: 78 pm), Mn^2+^ (HS: 83 pm), Sn^2+^ (122 pm), and Pb^2+^ (119 pm) are larger than the bond length of the H_2_ molecule, 74.1 pm.^[^ ^34^ ^]^ The H_2_ molecules adsorbed on such large ions are subjected to a weak electric field, which may lead to a sluggish O/P conversion similarly occurring over the metal catalysts. The radii of the high‐valence metal ions are generally smaller than those of the low‐valence ions. Indeed, V^5+^ (ionic radius: 54 pm), Sb^5+^ (ionic radius: 60 pm), and Ta^5+^ (ionic radius: 64 pm) are smaller than the corresponding low‐valence ions, V^3+^ (ionic radius: 64 pm), Sb^3+^ (ionic radius: 76 pm) and Ta^3+^ (ionic radius: 72 pm), respectively. The polarization of H_2_ molecules on the high‐valence metal surface is large when the molecules are adsorbed, pointing the side to the surface (side‐on adsorption), subjected to a strong, anisotropic electric field. High‐valence metal oxides such as V^5+^_2_O_5_, Sb^5+^_2_O_5_, Ta^5+^_2_O_5_, and Zr^4+^O_2_ exhibit prominent O/P conversion activity due to the promoted nuclear spin transition by the electric field.

**FIGURE 8:**
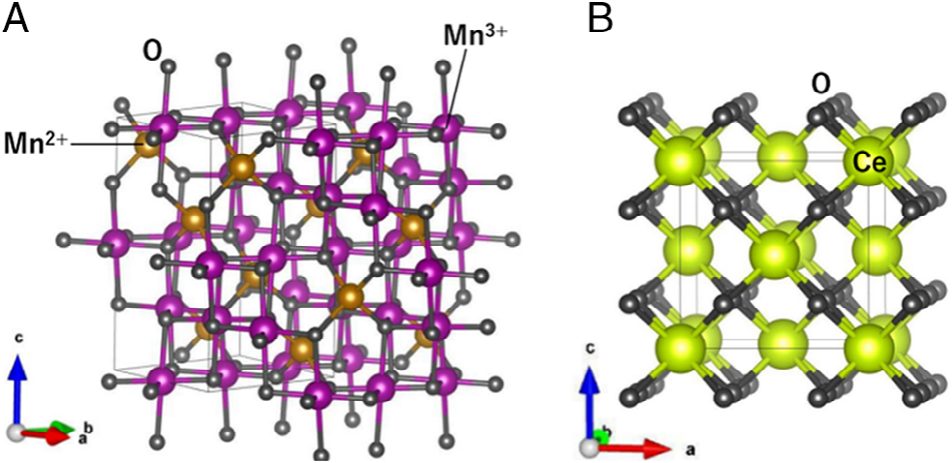
(A) Crystal structure of Mn_3_O_4_ and (B) CeO_2_. Mn_3_O_4_ adopts a distorted spinel‐type crystal structure.^[^ ^36^ ^]^ The cationic distribution of Mn^2+^/Mn^3+^ obeys normal spinel type. The Mn^3+^O_6_ octahedron shows Jahn‐Teller distortion, inducing symmetry lowering from cubic to tetragonal cells. CeO_2_ takes a fluorite‐type crystal structure.

Second, we discuss the character of group 4 where the magnetic field plays a crucial role. The nuclei of H_2_ molecules are affected not only by electric fields but also by magnetic interactions with the local moments that are distributed over the catalyst surface.^[^ ^6^, ^24^ ^]^ Indeed, the O/P conversion of hydrogen nuclei is promoted by a magnetic dipole‐dipole coupling with the electrons of hydrogen molecules in direct contact with electrically non‐polar matter such as oxygen molecules.^[^ ^35^ ^]^ However, the present experimental results show none of the magnetically metallic materials exhibits finite O/P activity, although some of the metals, such as Fe (Co, Ni) show inherently magnetism. Thus,magnetic interactions appear less predominant than electric interactions judging as a whole. Prominent O/P conversions are realized only if both the electric‐ and magnetic interactions are constructively applied to the H_2_ molecules, that is, in the case of group 4 materials including Mn_3_O_4_. Mn_3_O_4_ crystallizes in the normal spinel structure (Figure 8A).^[^ ^36^ ^]^ The Mn^3+^ cation is coordinated by six oxygen atoms to form a MnO_6_ octahedron. The octahedrally coordinated Mn^3+^ cation is so small in ionic radius that it is likely able to apply an anisotropic electric field to the H_2_ molecule and provide an opportunity for magnetic exchanges between the H_2_ nuclei and metal *d*‐electrons to accelerate the O/P conversion.

The same scenario may be valid for the other magnetic materials belonging to group 3, where the metal ions are allowed to strongly interact with the nuclei of H_2_ molecules such as Co(OH)_2_, Cr(OH)_3_, Fe_2_O_3_, or FeOOH. This effect seen in magnetic insulators obeys Wigner's theory.^[^ ^6^ ^]^ Mn^2+^Mn^3+^_2_O_4_ contains both Mn^2+^ (3*d* ^5^ HS electronic configuration) and Mn^3+^ (3*d* ^4^ HS) ions. The effective moment spin‐only value, μ_eff_ is given by 2[S(S+1)]0^1/2^, where S is the total spin of the ion. The μ_eff_ of *d* ^5^ or *d* ^4^ configuration is 5.92 or 4.90 BM, respectively. Mn^2+^ ions cannot satisfy the criterion of cationic radius described above, despite the large μ_eff_ (5.92 BM). On the other hand, the Mn^3+^ ion having a smaller ionic size contributes to the magnetic interaction through a moderately large μ_eff_ (4.90 BM), even if it undergoes Jahn‐Teller distortion leading to the buildup of a large anisotropic electric field. Finally, mentioned are some exceptional cases in our classification. According to the observation in Figure 5, there seems to be another group of materials in group 4. This group consists of CeO_2_ and gadolinium oxide (Gd_2_O_3_). Figure 8B shows the crystal structure of CeO_2_. It adopts a fluorite‐type crystal structure in which the Ce ion is surrounded by eight O ions (and we can see a cavity surrounded by eight O ions). They showed similar O/P conversion trends as the group 4 materials, such as Mn3O4, realizing 50% of the para‐H_2_ fraction within 20 min (Figure 4). However, the ionic radii for Ce^4+^ and Gd^3+^ being 87 pm and 94 pm, respectively,^[^ ^34^ ^]^ are significantly larger than the bond length of H_2_, 74.1 pm. The H_2_ molecules likely receive sufficiently strong electric fields from the surface of neither Ce^4+^ nor Gd^3+^ ions.

**FIGURE 9:**
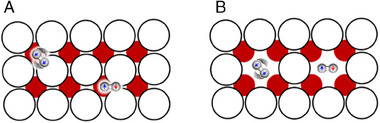
Adsorption of ortho‐ and/or para H_2_ onto the (A) metal ions and (B) oxygen vacancies. Black‐open circles and red‐closed circles denote oxygen (O^2−^) and metal ions, respectively.

It is acknowledged that lanthanide oxides can contain high concentrations of oxygen vacancies in the bulk and/or on the surface, in particular when the lanthanide ions are capable of adopting different valences. As‐synthesized CeO_2_ materials often contain trivalent Ce^3+^ ions, being accompanied by equivalent oxygen vacancies. The charged oxygen vacancy may adsorb H_2_ molecules in a similar way as the metal cations (Figure 9). Ortho H_2_ molecules are efficiently converted to para H_2_ before leaving the surface due to the anisotropic electric field at the oxygen vacancies. This scenario is supported by the experimental fact that a solid solution of CeO_2_ and Gd_2_O_3_ (Ce^4+^_0.8_Gd^3+^_0.2_O_1.9_) containing a high concentration of oxygen vacancy exhibits prominent O/P conversion activity. Artificially impregnated oxygen vacancies in Ce^4+^_0.8_Gd^3+^_0.2_O_1.9_ most likely play the role of a catalysis center.

## 5 SUMMARY

We have screened a wide range of materials to explore effective catalysts for the O/P conversion of H_2_ at 77 K. The primary conclusions are summarized as follows: (1) The O/P conversion catalysts are categorized into four groups. (2) The first is a group of materials that exhibit no finite activity toward the O/P conversion (group 1). This group involves all of the metallic materials and most of the oxides containing low‐valence metal cations. (3) Group 2 consists of metal oxides that contain high‐valence cations, such as V^5+^_2_O_5_. The materials exhibited finite O/P conversion activity: the para H_2_ fraction monotonously increased from 25% showing a tendency to saturate toward 50%. Most of the group 2 oxides comprise non‐magnetic metal ions without *d*‐electrons (V^5+^, Y^3+^, Ta^5+^, Sb^5+^). (4) The group 3 materials, such as Mn_2_O_3_ or CuO are similar in the ionic and catalytic nature to the group 2 materials yet show superior catalytic activity. (5) Group 4 involves some of the metal oxides and hydroxides that consist of magnetic metal cations. Mn_3_O_4_ and CoO showed much higher catalytic activity among the four groups. (6) Although there are various factors to influence the catalytic activity, the surface electric field in ionic compounds seems to be the most important one, which induces the electric polarization of adsorbed H_2_.

We can easily estimate this effect considering the cationic size in metal oxides: the oxides containing smaller cations (ionic radius < 75 pm) tend to show the activity empirically. This effect appears to work well for MOF materials but not for metallic materials with itinerant electrons. (7) Magnetism induced by open‐shell transition metal cations in the oxide catalysts enhances the catalytic activity through the theory proposed by Wigner. The activity of ionic oxide semiconductors, including Mn_3_O_4_ and CoO originates from both electric fields and magnetic interactions. (8) CeO_2_ also shows high activity without magnetic ions. It contains a large Ce^4+^ ion (size > 75 pm) and does not satisfy the criterion about cationic size. While we cannot find the primary factor for this oxide, the oxygen vacancy created as a result of the formation of Ce^3+^ would give rise to a large and anisotropic electric field on the surface. (9) There is a class of materials for which the activity change is distinct, just doping the foreign ions, but cannot be understood at this stage. While further efforts are needed to solidify the scientific base, these findings obtained through material exploration would be useful to design the optimal catalyst. It was unexpected for us that the electric field effect was more dominant than the magnetic interaction. This finding is quite consistent with the inertness of metallic materials. The electric field gradient over H_2_ adsorbed on the material surface appears to be a critical factor for effective O/P conversion catalysis. The results of the present exploratory research imply the conversion would be controlled by enhanced spin‐orbital interaction^[^ ^23^ ^]^ or nuclear quadrupole interaction.

## CONFLICT OF INTERESTS STATEMENT

The authors declare no conflicts of interest.

## Supporting information

Supporting Information

## Data Availability

The data that supports the findings of this study are available in the supplementary material of this article.

## References

[1] L. Barreto , A. Makihira , K. Riahi , Int. J. Hydrogen Energy 2003, 28, 267.

[2] W. McDowall , M. Eames , Energy Policy 2006, 34, 1236.

[3] F. Zhang , P. Zhao , M. Niu , J. Maddy , Int. J. Hydrogen Energy 2016, 41, 14535.

[4] G Nazir , A. Rehman , S. Hussain , S. Aftab , K. Heo , M. Ikram , S. Patil , M. A. U. Din , Adv. Sustainable Syst. 2022, 6, 2200276.

[5] S. Ghafri , S. Munro , U. Cardella , T. Funke , W. Notardonato , J. M. Trusler , J. Leachman , R. Span , S. Kamiya , G. Pearce , A. Swanger , E. D. Rodriguez , P. Bajada , F. Jiao , K. Peng , A. Siahvashi , M. Johns , E. May , Energy Environ. Sci. 2022, 15, 2690.

[6] E. Wigner , Z. Phys. Chem. 1933, B23, 28.

[7] Y. Ishi , S. Sugano , Surf. Sci. 1983, 127, 21.

[8] E. Ilisca , Prog. Surf. Sci. 1992, 41, 217.

[9] L. Farkas , Usp. Fiz. Nauk, 1935, 15, 347.

[10] A. Zhuzhgov , O. Krivoruchko , L. Isupova , O. Mart'yanov , V. Parmon , Catal. Ind. 2018, 10, 9.

[11] D. Ashmead , D. Eley , R. Rudham , J. Catal. 1964, 3, 280.

[12] P. W. Selwood , J. Am. Chem. Soc. 1966, 88, 2676.

[13] C. Ng , P. W. Selwood , J. Catal. 1976, 43, 252.

[14] Ionex Type OP Catalyst, https://www.molecularproducts.com/products/ionex‐type‐op‐catalyst (accessed: 12/5/2023).

[15] D. H. Weitzel , W. Loebenstein , J. Draper , O. Park , J. Res. Nat. Bur. Std. 1958, 60, 221.

[16] R. Svadlenak , A. Scott , J. Am. Chem. Soc. 1957, 79, 5385.

[17] R. A. Buyanov , Kinet. i Kataliz 1960, 1, 306.

[18] R. A. Buyanov , Kinet. i Kataliz 1960, 1, 418.

[19] M. Matsumoto , J. Espenson , J. Am. Chem. Soc. 2005, 127, 11447. 16089474 10.1021/ja0524292

[20] T. Das , S. Kweon , J. Choi , S. Y. Kim , I.‐H. Oh , Int. J. Hydrogen Energy 2015, 40, 383.

[21] J. H. Kim , S. W. Kang , I. W. Nah , I.‐H. Oh , Int. J. Hydrogen Energy 2015, 40, 15520.

[22] O. Boeva , A. Odintzov , R. Solovov , E. Abkhalimov , K. Zhavoronkova , B. Ershov , Int. J. Hydrogen Energy 2017, 42, 22897.

[23] T. Sugimoto , K. Fukutani , Nat. Phys. 2011, 7, 307.

[24] K. Fukutani , T. Sugimoto , Prog. Surf. Sci. 2013, 88, 279.

[25] T. Kosone , A. Hori , E. Nishibori , Y. Kubota , A. Mishima , M. Ohba , H. Tanaka , K. Kato , J. Kim , J. Real , S. Kitagawa , M. Takata , Roy. Soc. Open Sci. 2015, 2, 150006. 26587262 10.1098/rsos.150006 PMC4632575

[26] D. Polyukhov , N. Kudriavykh , S. Gromilov , A. Kiryutin , A. Poryvaev , M. Fedin , ACS Energy Lett. 2022, 7, 4336.

[27] T. Sugimoto , K. Fukutani , Phys. Rev. Lett. 2014, 112, 146101. 24765990 10.1103/PhysRevLett.112.146101

[28] E. Ilisca , Phys. Rev. Lett. 1991, 66, 667. 10043868 10.1103/PhysRevLett.66.667

[29] E. Ilisca , Europhys. Lett. 2013, 104, 18001.

[30] B. Stoicheff , Can. J. Phys. 1957, 35, 730.

[31] H. Abe , C. Nishimura , Y. Nohara , N. Okura , C. Fukuhara , R. Watanabe , M. Akaishi , K. Toyoshiba , WO Patent PCT/JP2022/040546, 2022.

[32] S. Khan , A. Hussain , K. He , B. Liu , Z. Imran , J. Ambreen , S. Hassan , M. Ahmad , S. S. Batool , C. Li , J. Environ. Manage. 2021, 293, 112854. 34058449 10.1016/j.jenvman.2021.112854

[33] K. Baraik , A. Bhakar , V. Srihari , I. Bhaumik , C. Mukherjee , M. Gupta , A. Yadav , P. Tiwari , D. Phase , S. Jha , S. Singh , T. Ganguli , RSC Adv. 2020, 10, 43497. 35519712 10.1039/d0ra09128f PMC9058517

[34] R. D. Shannon , Acta Crystallogr. 1976, 32, 751.

[35] X. Zhang , T. Karman , G. Groenenboom , A. Avoird , Nat. Sci. 2021, 1, 10002.

[36] M. Bekheet , I. Svoboda , N. Liu , L. Bayarjargal , E. Irran , C. Dietz , R. Stark , R. Riedal , J. Solid State Chem. 2016, 241, 54.

