# Supplementary material for: Exploration of heterogeneous catalyst for molecular hydrogen ortho‐para conversion
Source: Exploration (Beijing). 2023 Dec 14;4(3):20230040. doi: 10.1002/EXP.20230040 (PMC11189567; doi:10.1002/EXP.20230040)
Supplement: Supplementary file 1 — Supporting Information [file EXP2-4-20230040-s001.docx]

Supporting Information

**Exploration of Heterogeneous Catalyst for Molecular Hydrogen Ortho-Para Conversion**

Hideki Abe^1,2, *^, Hiroshi Mizoguchi^3^, Ryuto Eguchi^4^ and Hideo Hosono^3,5, *^

1. Center for Green Research on Energy and Environmental Materials, National Institute for Materials Science (NIMS), 1-1 Namiki, Tsukuba, Ibaraki 305-0044, Japan
2. Graduate School of Science and Technology, Saitama University, 255 Shimo-Okubo, Saitama 338-8570, Japan
3. Research Center for Materials Nanoarchitectonics (MANA), National Institute for Materials Science (NIMS),1-1 Namiki, Tsukuba, Ibaraki 305-0044, Japan
4. Faculty of Pure and Applied Sciences, University of Tsukuba, 1-1-1 Tennoudai, Tsukuba, Ibaraki 305-8573, Japan
5. MDX Research Center for Element Strategy, International Research Frontiers Initiative, Tokyo Institute of Technology, 4259 Nagatsuta, Midori-ku, Yokohama 226-8503, Japan


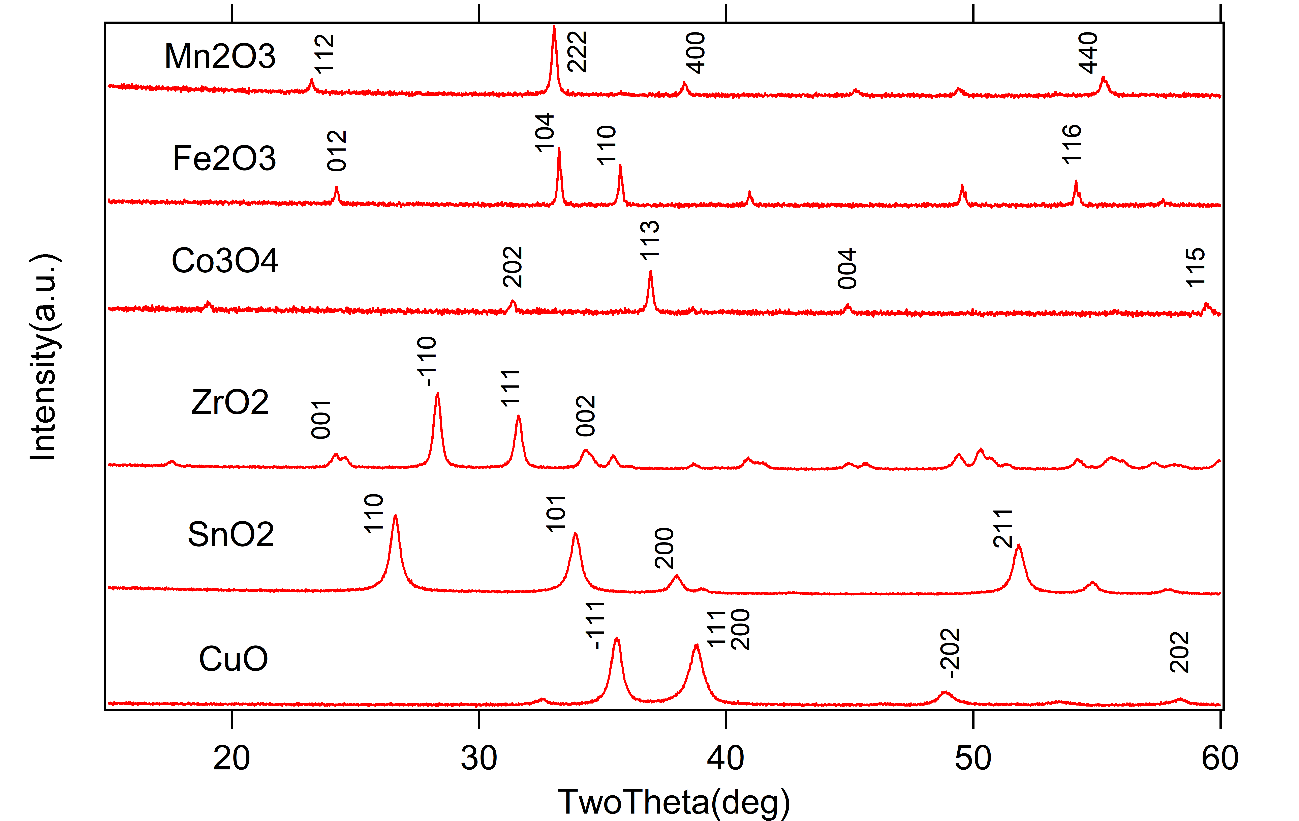


**Figure S1.** Powder X-ray Diffraction patterns for the catalysts belonging to the group 3.


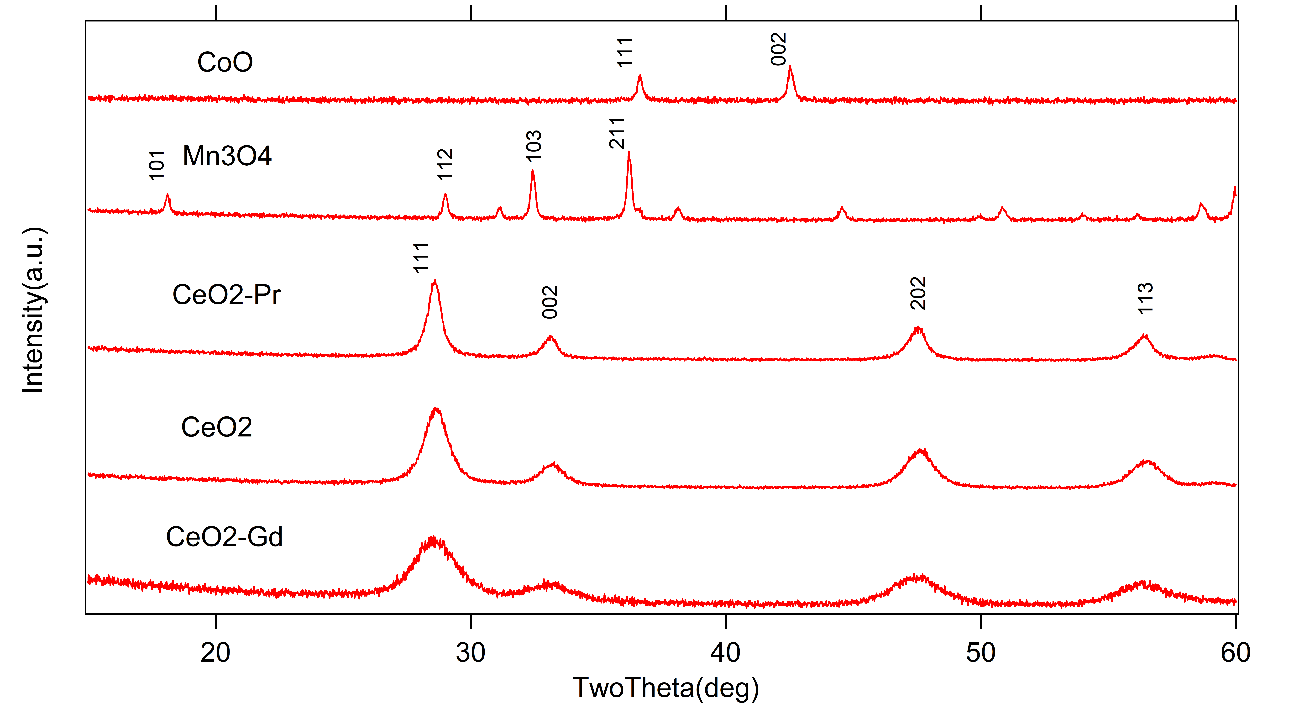


**Figure S2.** Powder X-ray Diffraction patterns for the catalysts belonging to the group 4.


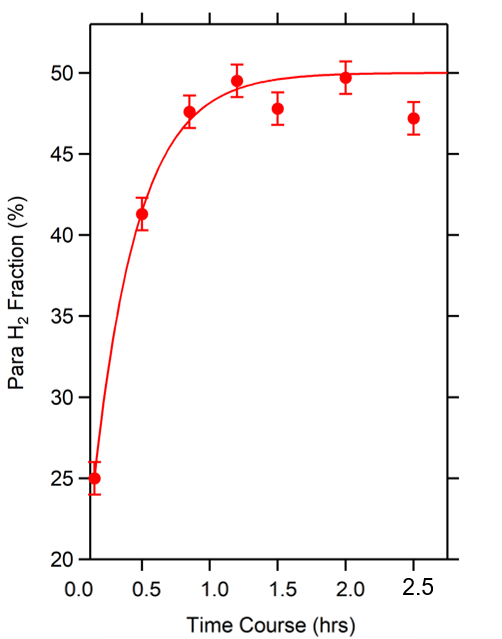


**Figure S3.** Exponential fitting to the experimentally obtained data for a Mn_3_O_4_ catalyst material. The exponential function is defined as W_coef[0] - W_coef[1] x exp(-1 x W_coef[2] x Time Course, where W_coef[0] = 50 (constant), W_coef[1] = - 8.55±1.52 and W_coef[2] = 3.07±0.53 (h^-1^). The rate constant for a given catalyst corresponds to W_coef[2].
